# The Era of Resistance Training as a Primary Form of Physical Activity for Physical Fitness and Health in Youth Has Come

**DOI:** 10.1007/s40279-025-02240-3

**Published:** 2025-05-26

**Authors:** Helmi Chaabene, Rodrigo Ramirez-Campillo, Jason Moran, Lutz Schega, Olaf Prieske, Ingo Sandau, Yassine Negra, Martin Behrens

**Affiliations:** 1 Department of Sport Science, Chair for Health and Physical Activity, Otto-von-Guericke University Magdeburg, Magdeburg, Germany; 2 Institut Supérieur de Sport et de l’Education Physique du Kef, Université de Jendouba, 7100 Le Kef, Tunisie; 3 Exercise and Rehabilitation Sciences Institute, School of Physical Therapy, Faculty of Rehabilitation Sciences, Universidad Andres Bello, 7591538 Santiago, Chile; 4 Sport Sciences and Human Performance Laboratories, Instituto de Alta Investigación, Universidad de Tarapacá, Arica, Chile; 5 School of Sport, Rehabilitation and Exercise Sciences, University of Essex, Colchester, Essex, UK; 6 University of Applied Sciences for Sport and Management Potsdam, Olympischer Weg 7, 14471 Potsdam, Germany; 7 Department Strength, Power and Technical Sports, Institute for Applied Training Science, Leipzig, Germany; 8 Research Unit (UR17JS01) Sports Performance, Health & Society, Higher Institute of Sport and Physical Education of Ksar Saïd, University of Manouba, 2010 La Manouba, Tunisia; 9 Department of Orthopaedics, Rostock University Medical Center, Rostock, Germany

## Abstract

Resistance training (RT) is widely regarded as the gold standard approach for enhancing muscular fitness (i.e., muscle strength, power, and muscular endurance) in youth while also providing health and physical fitness benefits traditionally associated with aerobic training (e.g., enhanced cardiorespiratory fitness, reduced body fat, improved insulin sensitivity). Additionally, while bone health can be improved following RT (particularly after plyometric jump training), aerobic training may result in a lesser or even neutral impact on bone mineral density enhancement (e.g., swimming). Regarding mental health and cognition, while aerobic training has well-established positive effects, preliminary evidence in obese youth suggests that RT may offer greater benefits in certain aspects compared to aerobic training. Additionally, RT can reduce the risk and incidence of injuries in youth. Overall, we argue in this Current Opinion article that the current consideration of RT as an additional, rather than essential (possibly even the most essential), aspect of physical activity in current national and international guidelines needs to be reconsidered. Overall, there is an urgent need to inform relevant stakeholders that, while aerobic activities remain essential, the next generation of physical activity guidelines should place greater emphasis on the particular importance of RT, providing more comprehensive guidance on its implementation for youth.

## Key Points

**Table:** 

The current physical activity guidelines, which state that youth should engage in at least an average of 60 min of moderate-to-vigorous, mostly aerobic physical activity daily with vigorous-intensity aerobic activities, as well as muscle and bone strengthening exercises, carried out at least three times weekly as part of the 60 min of daily activity, seem to emphasize aerobic over muscle strengthening exercises (i.e., resistance training).
Promoting resistance training is paramount to combat the widespread effects of physical inactivity, improving muscular fitness, and reducing activity-related injuries as well as adverse health events in youth.
Resistance training can also yield adaptations typically associated with aerobic training, such as enhanced cardiorespiratory fitness, reduced body fat, and increased insulin sensitivity. Indeed, while aerobic training alone cannot entirely replace resistance training in youth, resistance training appears to provide some level of substitution.
There is an urgent need to update the current hierarchy of physical activity guidelines for youth by prioritizing resistance training and providing clearer recommendations on dosage. However, this shift would not minimize the benefits of aerobic training, particularly the potential synergistic effects of combining resistance and aerobic training.

## Introduction

After decades of questioning the safety and efficacy of resistance training (RT) in youth (an umbrella term for children and adolescents [1]) [2], extensive and persuasive evidence to the contrary has now emerged, starting with a seminal review by Kraemer et al. [3] and subsequent publications [4–14]. Of note, RT is a specialized method of physical conditioning that involves the progressive application of varying resistive loads, movement velocities, and training modalities including equipment such as weight machines, free weights (barbells and dumbbells), elastic bands, medicine balls, and plyometric^1^^[15]^ exercises [6]. There is now a widespread consensus that RT is safe, effective, and an irreplaceable tool to promote both physical fitness^2^^[16]^ and health in youth. Indeed, the benefits of RT cover a wide range of health aspects such as strength [17, 18], bone mass/bone mineral density (BMD) [19–24], body composition [23, 25–28], mental health [23, 26], and cognitive function [26, 29]. Additionally, RT may aid in preventing negative health outcomes in youth such as type 2 diabetes [24, 26, 30], cardiovascular diseases [23, 26, 31, 32], as well as premature death [33]. Furthermore, it is well known that RT improves muscular fitness (i.e., muscle strength, muscle power, and muscular endurance) in youth [25, 34–36]. However, despite the remarkable, well-established benefits of RT, the adherence rate to physical activity (PA), including RT, among youth is alarmingly low. Evidence derived from a pooled analysis of 298 population-based surveys including 1.6 million participants indicates that 81% of youth aged between 11 and 17 years fail to adhere to the World Health Organization (WHO)’s PA guidelines and are thus classified as physically inactive [37]. This is consistent with other studies including those with large sample sizes [38, 39]. This reflects a widespread global trend of physical inactivity^3^ and sedentary behaviour among youth [40]. Given the well-established tracking character of PA and muscular fitness [24, 41–43], not only short but also long-term negative consequences on physical fitness and health can be expected.

Established public health institutions, including the United States Department of Health and Human Services [23] and the WHO [44], communicate recommendations for PA to the public. These guidelines represent a crucial source of information for youth themselves, their parents, policy-makers, and healthcare professionals [9]. As such, the accuracy of such guidelines with respect to the available evidence is of utmost importance to promote healthy behaviours [45]. The most up-to-date PA guidelines by the WHO indicate that youth should engage in at least an average of 60 min of moderate-to-vigorous, primarily aerobic^4^ PA daily [44]. Additionally, within these 60 min, vigorous-intensity aerobic activities as well as muscle- and bone-strengthening exercises should be carried out at least three times weekly [44]. In particular, these recommendations emphasize the amount of aerobic PA that should be accumulated across the week. However, the early development of muscle strength in youth is of utmost relevance [46]. It is noteworthy that a low level of muscle strength in youth, referred to as paediatric dynapenia, can have severe consequences pertaining to the development of functional limitations (i.e., impaired fundamental movement skills^5^^[47]^), physical inactivity and sedentary behaviour, as well as an increased risk of negative health events (e.g., higher risk of injuries and chronic diseases) [4, 7, 10, 12].

While the significance of aerobic training in preventing chronic diseases in youth is undeniable [23], RT can be equally effective as aerobic training, such as in preventing type 2 diabetes [48, 49]. For instance, Grøntved et al. [48] investigated the association of muscle strength and cardiorespiratory fitness with indices of glucose metabolism in healthy youth, followed over 12 years. The findings indicated similar levels of association between muscle strength and cardiorespiratory fitness with both insulin resistance and β-cell function. The authors concluded that muscle strength is similarly effective as cardiorespiratory fitness in preserving healthy insulin sensitivity and β-cell function later in life [48]. Notably, these associations were independent of adiposity and demographic, personal, and lifestyle factors [48]. Therefore, early engagement in aerobic or RT substantially and similarly reduces the risk of impaired insulin sensitivity and improves β-cell function in later years. Lee et al. [49] examined the effects of three months' RT versus aerobic training on abdominal adiposity, ectopic fat, and insulin sensitivity in obese male youth. Their findings revealed similar reductions in abdominal fat and intrahepatic lipid across both training modalities, but RT (unlike aerobic training) was associated with significant improvements in insulin sensitivity. Additionally, there are indications that the adherence rate differs between RT and aerobic training in youth. Indeed, 50.7% met the guidelines for muscle-strengthening (e.g., exercises to strengthen or tone the muscles such as push-ups, sit-ups, and weight lifting), 70.6% met the vigorous aerobic PA guidelines, and 80.7% met those for bone-strengthening, but overall only 15.2% adhered to the moderate-to-vigorous, mostly aerobic PA guidelines [50] as per the United States Physical Activity guidelines [51]. These findings are supported by other studies [52, 53]. However, caution is warranted when interpreting these results, as differing standards are applied to assess adherence to each type of PA. Specifically, meeting the criterion of engaging in RT at least 3 days per week is considerably easier than achieving an average of 60 min of aerobic moderate-to-vigorous PA daily. Furthermore, weaker youth often lack the ability, confidence, and motivation to engage in exercise and sports activities, increasing the likelihood of adopting a sedentary behaviour/lifestyle [7, 24, 54–56]. Early exposure to RT has the potential to counteract this trend by fostering strength, confidence, and competence. This foundation can enable youth to engage in and sustain regular PA, thereby improving adherence to PA guidelines [7, 9, 56]. Therefore, RT could be considered a more effective approach than aerobic training, particularly in helping to mitigate the widespread issue of physical inactivity among youth. Another potential advantage of RT is that it may be more preferred than aerobic training. Evidence suggests that RT is more enjoyable than aerobic training (e.g., running, cycling) in youth, particularly those who are overweight or obese [25, 57, 58]. While direct comparisons in healthy youth are lacking, we speculate that similar outcomes may be observed in this population.

In this Current Opinion article, we argue that the current PA recommendations’ hierarchy that emphasizes aerobic over RT needs to be revised. Specifically, we call for reversing the current priority framework by prioritizing RT, without disregarding the complementary effects of combining it with aerobic training. Previous review papers have underscored the importance of RT in adults and older adults [8, 59]. In youth, however, the sole available study by Faigenbaum et al. [9] emphasized the significance of RT in youth for enhancing physical fitness and health, and advocated for prioritizing muscular fitness development in youth guidelines. Nonetheless, a comprehensive description of the health benefits of RT is still lacking, and studies comparing aerobic training and RT have not been thoroughly summarized. Therefore, this Current Opinion article aims to synthesize the available evidence regarding the effects of RT compared to aerobic training on physical fitness and health outcomes in youth and to advocate for greater emphasis on RT for youth in the next generation of PA guidelines.

## The Compound Threat of Paediatric Dynapenia

Paediatric dynapenia is one of the leading contemporary threats to health among youth [7, 10, 60, 61]. The term dynapenia has been adopted from the literature relating to geriatric patients and stands for decreased levels of muscle strength and power not caused by neurological or muscular disease [10, 61], meaning that paediatric dynapenia is identifiable and treatable. The alarmingly low adherence rate to PA among youth worldwide [37–39] appears to be the main driver of the secular decline in measures of muscular fitness and, therefore, dynapenia [62–64]. In a recent study investigating the secular trend of physical fitness among a large sample of Chinese rural youth, Li et al. [62] reported increased muscle strength and power from 1985 to 2000. However, this positive trend has reversed from 2000 to 2010, marked by a decline in muscle strength and power. Notably, this decline persisted through the subsequent period from 2010 to 2019 [62]. In fact, the secular decline in muscular fitness among youth has been consistently reported by other studies [65–69], though this trend does not apply to all measures of physical fitness, such as handgrip strength [70]. Indeed, a 3-year longitudinal study demonstrated that physical fitness, including muscular fitness, is significantly correlated with the time spent in moderate-to-vigorous PA in youth [64]. Additionally, a systematic review of the literature by Smith et al. [71] supported the positive correlation among youth between PA and muscular fitness, specifically vigorous PA and organized sports participation. Recently, Fraser et al. [72] revealed that PA is one of the key factors leading to better muscle strength in the long term.

Physical fitness, particularly muscle strength, is considered a powerful marker of health [19, 24, 73–76]. Cumulative evidence suggests that the current generations of youth are weaker than previous ones [62, 65–67], indicative of a growing trend in paediatric dynapenia. It is worth noting that the relationship between paediatric dynapenia (muscle weakness) and PA is bidirectional. While we acknowledge that paediatric dynapenia generally develops as a result of physical inactivity in healthy youth, there are also cases when dynapenia precedes physical inactivity, particularly in unhealthy youth. For instance, congenital or neuromuscular disorders in youth may result in primary muscle weakness, leading to reduced PA [77–79]. Once it occurs, paediatric dynapenia leads to a cascade of severe negative consequences, including increased functional limitations and decreased fundamental movement skills in youth [7, 9, 10, 54, 60, 61]. Weaker youth may lack the ability, confidence and motivation to engage in exercise and sports activities, increasing the likelihood of adopting a sedentary behaviour [7, 24, 54, 55]. This tendency is further accentuated by the availability of sedentary alternatives like screen time and video games [9, 43, 80]. As a result, these youths are more exposed to adverse health outcomes caused by muscle disuse and physical inactivity [81, 82], a trend that has been demonstrated in numerous studies [31, 83]. Earlier studies have shown that muscle strength in adolescent males is inversely correlated with cardiovascular disease events and mortality in middle age, regardless of cardiorespiratory fitness and other confounding factors such as smoking and alcohol consumption [31]. Moreover, in a large cohort of 1.2 million males, Henriksson et al. [83] revealed that muscle weakness is associated with disability 30 years later.

Persuasive evidence indicates that PA [84–87], physical fitness [41, 42], and more particularly muscle strength [42, 88] track from childhood to adulthood. Therefore, it is plausible to argue that paediatric dynapenia also tracks from early to later ages. Fraser et al. [88] investigated the track of muscle strength from youth to adulthood in a sample of 1207 participants and reported a significant tracking correlation ranging from 0.47 to 0.72. The results of another prospective cohort study including 623 participants corroborate the previous findings indicating a relatively stable muscle strength and power between youth and adulthood with a tracking correlation ranging from 0.43 to 0.47 [42]. Additionally, inactive youth tend to become resistant to exercise interventions later in life [89], emphasizing the utmost need for structured and systematic programs aiming at combating strength deficits in youth. This needs to be undertaken at an early age to develop a routine pattern of healthy PA behaviour, including RT, that will persist to later stages in life. Evidence suggests that RT can be started from the age of 7–8 years, or even earlier, depending on individual readiness, provided the youth can understand/follow instructions and is able to demonstrate proper movement technique [7, 14, 90, 91]. Key considerations when introducing RT to youth include prioritizing technique, supervision by qualified trainers, and ensuring the program is age-appropriate [90, 92, 93].

Taken together, paediatric dynapenia must be actively prevented and early proactive measures should be undertaken to avoid its occurrence. Current public health recommendations for youth advocate engaging in at least an average of 60 min of moderate-to-vigorous, mostly aerobic PA daily, with vigorous-intensity aerobic activities as well as muscle- and bone-strengthening activities incorporated at least 3 days a week as part of the 60-min activity [44]. However, these guidelines primarily emphasize the amount of aerobic PA, while placing less focus on the critical importance of muscle-strengthening exercises. It is crucial to recognize that promoting muscle strength through RT is paramount for combating physical inactivity, improving fundamental movement skills, and reducing activity-related injuries as well as adverse health events. As such, while we acknowledge the importance of aerobic training, there is a pressing need to shift the focus in the next generation of PA guidelines towards RT and to provide more detailed guidelines related to its dosage.

## The Effects of Resistance Training versus Aerobic Training on Health Outcomes in Youth

### Chronic Diseases

Recently, Brellenthin et al. [59] indicated that RT is as beneficial as aerobic training for several health issues such as type 2 diabetes, cancer, cardiovascular disease, and obesity, among adults. The same authors emphasized that individuals who perform both RT and aerobic training would experience the greatest health benefits, reflecting a synergistic effect on health-related outcomes [59]. In youth populations, studies that directly contrasted the association of RT and aerobic training with key health outcomes are scarce [49, 94, 95]. Additionally, most of the studies compared the association of RT and aerobic training with changes in health outcomes such as insulin sensitivity and body fat in overweight and obese youth [49, 95–98]. For example, Lee et al. [49] investigated the effects of 3 months of RT versus aerobic training on abdominal adiposity, ectopic fat, and insulin sensitivity in obese male youth, revealing similar reductions in abdominal fat and intrahepatic lipid, although only RT was associated with marked improvements in insulin sensitivity. In a randomized controlled trial, Sigal et al. [97] compared RT, aerobic training, and combined training effects on body composition and cardiometabolic risk factors in overweight and obese youth, revealing similar decreases in percentage body fat and waist circumference following RT and aerobic training. However, combined RT and aerobic training exerted a synergistic effect on the same outcomes [97]. In a similar study in overweight and obese adolescents, Lee et al. [98] examined the effects of RT, aerobic training, or combined training on insulin sensitivity, total adiposity, and ectopic fat in overweight and obese adolescents and reported that all training modalities were beneficial in reducing body fat and intermuscular adipose tissue, as well as enhancing insulin sensitivity. They specifically reported that combined RT and aerobic training as well as aerobic training alone were similarly effective in reducing ectopic fat in the liver and skeletal muscle [98]. Moreover, these training modalities also increased insulin sensitivity, but aerobic training was relatively more effective by approximately 41% than RT regarding the improvement of insulin sensitivity [98]. Inoue et al. [99] conducted an intervention study including obese youth and concluded that combined RT and aerobic training was more effective than aerobic training alone to improve lipid profile and insulin sensitivity. In the same context, Goldfield et al. [94] contrasted the effects of 22 weeks of RT versus aerobic training versus combined training on health-related quality of life (measured using the Paediatric Quality of Life Questionnaire) in overweight and obese youth of both sexes. The results indicated that both RT and aerobic training alone improved health-related quality of life but that combined RT and aerobic training generated the largest improvements relative to the control group. Furthermore, Dâmaso et al. [100] demonstrated that 1 year of combined RT and aerobic training was more effective than aerobic training alone in improving visceral adiposity, metabolic profile, and inflammatory markers in obese youth. Moreover, in a critical summary of the available evidence, Lee et al. [101] concluded that single-mode RT is associated with a significant decrease in total fat and insulin resistance in previously sedentary obese youth. In summary, accumulating evidence indicates that the health benefits of RT are similar to those of aerobic training concerning total adiposity, cardiometabolic risk, and insulin resistance. Additionally, the combination of both training modalities seems to provide synergistic effects in overweight and obese youth.

In healthy youth, however, investigations that directly compared the impact of RT, aerobic training, or combined training on important health-related outcomes are scarce. Emerging evidence indicates that muscle strength is also associated with cardiovascular health [31, 102]. Specifically, muscle strength in adolescent males is inversely associated with later cardiovascular disease events and mortality in middle age, independent of cardiorespiratory fitness and other confounding variables (e.g., smoking, alcohol consumption) [31]. Additionally, Åberg et al. [102] conducted a prospective cohort study including ~ 1.5 million Swedish male conscripts over 42 years. The study aimed to examine whether aerobic fitness or muscle strength registered at a young age (18 years), independently or combined, correlate with long-term stroke risk [102]. Low aerobic fitness in youth and high stroke incidence during adulthood were noted, with a hazard ratio (HR) of up to 1.70, i.e. 70% increased risk for those with low aerobic fitness at a young age [102]. However, the investigators also revealed that low muscle strength in youth was associated with increased stroke incidence during adulthood, with an HR of 1.39 (1.17 after accounting for aerobic fitness) in the low muscle strength group compared with the high muscle strength group, pointing towards the independent nature of the association between muscle strength in youth and stroke risk later in life [102]. In a prospective cohort study including ~ 1 million male adolescents, Ortega et al. [33] revealed that those with a muscle strength performance equal to or above the 40th percentile of the studied population displayed a 20% lower risk of all causes of premature death and 25% lower risk of cardiovascular disease than those in the 10th percentile. Additionally, for each 5% decrease in muscle strength, there was a 1.48 increased odds of high cardiometabolic risk in youth males and a 1.45 increase in females [103]. Furthermore, Artero et al. [104] demonstrated that metabolic risk in youth was independently associated with both muscular and cardiorespiratory fitness. Other studies [105, 106] clearly indicate that a low level of muscle strength is one of the contributing factors to metabolic dysfunction in youth. Henriksson et al. [83] conducted a prospective cohort study with ~ 1.2 million participants to investigate the associations between muscle strength in adolescence with later disability pension. Disability pension is granted if an individual is likely to never work full-time again due to severe chronic disease or injury. The authors reported a strong association between muscular weakness and disability 30 years later [83]. They also found that the combination of low muscle strength and low aerobic fitness was a prominent risk factor for disability [83]. Furthermore, the findings indicated that being unfit, weak and obese exhibited the highest association with disability pension risk (HR = 3.70, 95% confidence interval (CI) = 2.99 to 4.58) [83]. Of note, correlation (e.g., prospective cohort studies) does not directly imply causation, and this should be taken into consideration [107].

Overall, compelling evidence suggests that low muscle strength results in adverse health outcomes for both the present and the future health of youth. These outcomes include an increasing risk of developing metabolic dysfunction, cardiovascular diseases, and insulin resistance [32, 97, 99, 102, 103, 106, 108–112]. More specifically, accumulating evidence indicates that RT generates beneficial effects on health outcomes traditionally attributed solely to aerobic training. While aerobic training might outperform RT in preventing cardiovascular diseases [102], both RT [48, 49] and aerobic training [48, 98] can lead to improved insulin sensitivity. Combining both training modalities appears to have synergistic effects on numerous health-related outcomes [48, 94, 97, 99, 100]. However, it is imperative to conduct additional high-quality studies that directly compare the effects of RT and aerobic training on key health outcomes, particularly in healthy youth. Given the limited literature in this area, this is essential to enhance our understanding. Furthermore, exploring the physiological mechanisms that underpin the link between RT and improvements in diverse health outcomes in youth presents significant opportunities for future research.

### Bone Health

Growing bones around puberty are more sensitive to mechanical loading compared to adults’ bones [113], making this age period optimal for improving bone health in youth [20, 114–116]. The benefits to bone health arise from the synergistic interaction between the natural growth-related increase in bone mass and mechanical loading, particularly induced by RT [20]. Indeed, the American College of Sports Medicine proposed two main strategies to maintain and/or improve bone health: (i) maximizing BMD during the first 30 years of life, and (ii) mitigating the decrease in BMD after 40 years [117]. Increasing BMD during childhood and adolescence is the most appealing strategy, as this will track into adulthood, reducing the risk of fractures at a later age [113, 114, 118–121]. Moreover, peak bone mass during puberty and early adulthood is a powerful predictor of the risk of osteoporosis in older age in females [119]. Therefore, given the track character of bone health, it seems imperative to implement effective interventions to enhance bone health from an early age. This proactive approach can help to mitigate the risk of osteoporosis and fractures later in life, especially in females [122].

Muscle strength and mass are considered key predictors of bone health [32, 123]. Sioen et al. [124] conducted a systematic review of observational and longitudinal studies that examined the correlation between muscle mass and bone parameters in youth. They reported that most investigations showed positive associations between muscle mass and BMD, bone mineral content (BMC), and bone area [124]. Additionally, a consistent positive relationship between muscle strength in youth and bone parameters has been reported in the literature [32]. For example, significant positive associations between handgrip strength and BMD as well as BMC at the hip, spine and entire body have been reported, regardless of children’s sex [125]. The same authors concluded that handgrip strength could be considered an independent predictor of BMD and BMC in children [125]. This conclusion was supported by other studies in youth [126–129].

Different types of RT, such as machine-based and free-weight RT, can benefit BMD in youth [114]. More particularly, high-impact or jump-based exercises that create sufficient ground reaction forces (GRFs) are considered the most prominent RT modality to promote osteogenic processes and improve bone health in youth [21, 113, 114, 117, 130–135]. More specifically, plyometric jump training may have the potential to maximize bone mineralization when applied during the pubertal growth spurt [136]. Indeed, jump-based exercises with GRFs ranging from 3.5 to 8.8 of one’s own body mass conducted for 10 min two to three times per week are effective in enhancing BMD among children and adolescents [137]. This suggests that for bone adaptations, the intensity of RT is a key factor and long-duration training sessions are not required. Gómez-Bruton et al. [138] conducted a systematic review of the effects of plyometric jump training on bone health in youth, and revealed that out of the 26 included studies, 24 demonstrated improved bone health, reflecting widespread consensus in the literature. The same authors concluded that plyometric jump training during childhood and adolescence has the potential to foster BMC, density BMD, and structural properties without side effects [138]. Ishikawa et al. [21] conducted a meta-analysis, and reported that high-impact weight-bearing activities (e.g., plyometric jump training) induced the largest benefits on bone mineral accrual in prepubertal females.

While aerobic PA could increase BMD [114, 139], the effects are generally lower than those generated by RT [114, 140, 141]. Furthermore, some types of aerobic exercise, specifically non-weight-bearing activities such as swimming and cycling may have a lesser or even neutral impact on BMD enhancement [114, 142–146]. Therefore, to optimize bone health, these activities should ideally be complemented by RT exercises designed to stimulate bone strength and density. This appears to be due to the relatively low mechanical stimulus during swimming and cycling, which is still below the threshold for positive osteogenic effects [128–130]. Ribeiro-Dos-Santos et al. [145] showed that the prolonged practice of swimming can negatively affect BMD gains in adolescents. Indeed, swimmers train in a hypogravitational environment where muscles do not have to counteract gravity, resulting in a low mechanical stress—a crucial factor that promotes BMD improvement [131, 132].

In summary, peak bone mass is typically attained during the developmental period, around puberty [115]. Therefore, improving bone health at an early age has a positive lifelong impact on future bone health. RT, particularly plyometric jump training, yields substantial positive effects on bone health in youth. Conversely, aerobic training appears to be less effective and may even have deleterious effects on bone health [114, 146].

### Mental Health/Cognitive Function

The results of a cross-sectional study aiming at investigating the independent and joint associations of RT and aerobic training with mental health in adolescents indicated that meeting either the RT or aerobic PA guidelines alone led to a lower prevalence of ever feeling sad or hopeless and difficulty making decisions [147]. Additionally, meeting both recommendations was more strongly associated with fewer mental health problems [147].

Goldfield et al. [57] conducted a randomized controlled trial on the effects of 6 months' RT versus aerobic training versus combined training versus no training on mood, self-esteem and body image of obese male and female adolescents. The authors revealed that only RT reduced depressive symptoms and that combined training led to greater improvement in vigour compared with the control group. Additionally, only RT improved global self-esteem and only RT and combined training enhanced perceived strength compared to the control group [57]. Therefore, RT offers psychological benefits among adolescents with obesity, thus presenting a potential exercise alternative for youth who find aerobic training uncomfortable or unenjoyable [57]. Recently, Chiang et al. [148] conducted a large cohort study including 1.9 million youth to examine the association between physical fitness and the risk of mental disorders. They reported that youth in higher fitness quantiles exhibited lower cumulative incidences of anxiety disorders, depressive disorders, and attention-deficit/hyperactivity disorder. Specifically, Chiang et al. [148] demonstrated that improvements in both muscular fitness, particularly muscular power and endurance (commonly achieved through RT), and cardiorespiratory fitness (typically resulting from aerobic training), were independently associated with reduced prevalence of mental health disorders in youth. The authors concluded that muscular and cardiorespiratory fitness could be considered protective factors against the onset of mental disorders in youth [148]. Eather et al. [149] conducted a randomized controlled trial to explore the impact of the CrossFit™ Teens RT on the mental health of adolescents, and revealed enhanced mental well-being in adolescents at risk of psychological disorders. Collins et al. [150] conducted a systematic review with meta-analysis on the effects of RT on the “self” (i.e., self-esteem, self-efficacy and self-perception) in youth, reporting improved RT self-efficacy, perceived physical strength, physical self-worth, and global self-worth. Furthermore, Robinson et al. [29] carried out a systematic review with meta-analysis investigating the effect of RT on academic outcomes in school-aged youth, and demonstrated that RT resulted in positive effects, although small in magnitude, on the combined outcomes of cognition, academic achievement, and on-task behaviour. In addition, findings indicated that RT was more effective than concurrent training (i.e., combined RT and aerobic training) and that higher levels of muscular fitness were associated with better performance in tests of cognition and academic achievements in the same population [29]. Albeit not well explored yet, there are indications that the increased cognitive demands of RT appear to support improvements in cognition and academic outcomes, potentially through mechanisms such as neurogenesis [151].

In summary, while the beneficial effects of aerobic training (particularly high-intensity interval training) on mental health and cognition in youth are undeniable [148, 152–155], preliminary evidence, primarily in obese youth, suggests that RT may offer greater benefits in certain aspects compared to aerobic training [57]. In particular, RT seems to be more enjoyable than aerobic training (e.g., running, cycling) in youth, especially for overweight and obese youth [25, 57, 58]. Future studies comparing the effects of RT and aerobic training on mental health and cognitive performance in youth are warranted to confirm the preliminary findings in obese youth and to explore their effects in healthy youth, a topic that has not been thoroughly investigated yet. Additionally, the neurophysiological mechanisms underlying RT-related cognitive adaptations in youth remain to be fully understood.

### Injury Prevention

RT is a well-established powerful injury prevention strategy in youth [6, 12, 54, 156, 157]. Generally, there is compelling evidence that youth with low levels of physical fitness, including muscle strength, and poor movement competency are more exposed to injuries [158–160].Torres Martín et al. [161] investigated the effects of 15 weeks of body mass-based RT on musculotendinous injury incidence and burden in U16 male soccer players. Albeit not significant, they reported a decreased musculotendinous injury incidence in the intervention compared to the control group (1.19 vs. 1.40 injuries/1000 h of exposure, respectively) [161]. Moreover, body mass RT significantly reduced injury burden, defined as the number of days lost per 1000 h of exposure (33.28 [control group] vs. 9.55 [intervention group]), indicative of a decreased severity of musculotendinous injuries in the RT compared to the control group [161]. Collard et al. [158] investigated the impact of a school-based PA program (encompassing strength, speed, flexibility, and coordination exercises) on injuries occurring during physical education sessions among primary school children aged 10–12 years. Their findings revealed a significant decrease in the rate of PA-related injuries, particularly notable among the least physically active children, with an HR of 0.47 (53% reduction in total injuries observed).

Neuromuscular training is an umbrella term that covers general (e.g., fundamental movement skills) and specific (e.g., sport-specific actions) strength and conditioning activities such as resistance, balance, core strength, plyometric and agility exercises [1]. The findings of a meta-analysis including 25 studies examining the effects of neuromuscular training reported ~ 36% reduction in lower limb incidence rate ratio in youth team sport [162], consistent with earlier reviews [163–166]. The preventive effects (68% risk reduction) of neuromuscular training on anterior cruciate ligament injuries in female athletes have also been highlighted in the meta-analysis by Sugimoto et al. [167]. Moreover, in another meta-analysis, Steib et al. [168] reported that neuromuscular training resulted in a 42% injury rate reduction in youth athletes.

In sum, RT and neuromuscular training are effective strategies to reduce the risk and rate of injuries in youth athletes as well as youth from the general population. The neural adaptations associated with RT (e.g., enhanced neural drive) and structural/mechanical changes (e.g., hypertrophy, increased musculotendinous stiffness) improve movement biomechanics, which is one of the key mechanisms underlying the reduced risk and incidence of injuries following RT [6]. Additionally, increased muscle strength and endurance, reflecting a higher fitness level, can help reduce the likelihood of fatigue-related injuries [169, 170]. Furthermore, enhanced joint stability, better balance and proprioception, and reduced muscular imbalances are key factors contributing to the reduction in injury risk among youth following RT [169–172]. RT is a widely recognized method to improve neuromuscular function,^6^ regardless of age [34, 92, 173–175]. Indeed, RT improves neural (e.g., motor unit recruitment and firing rate, intermuscular coordination) and structural (e.g., muscle hypertrophy) outcomes in youth [173, 176, 177]. Aerobic training, on the other hand, is a well-known means to improve cardiorespiratory fitness in youth [44, 178–180], although a certain effect on muscle hypertrophy cannot be totally ruled out [181, 182].

## The Effects of Resistance Training and Aerobic Training on Physical Fitness in Youth

### Muscular Fitness

Among the various training methods, none has proven as effective in enhancing muscular fitness, particularly muscle strength and power, in youth as RT [4, 5, 7, 9, 12, 44, 61, 173, 176]. Indeed, RT can improve measures of muscle strength and power in athletic [172, 183–191] and non-athletic populations [192–196]. For instance, Lesinski et al. [197] in a systematic review with meta-analysis of 43 original studies indicated that RT generated large effects on proxies of muscle strength in trained youth. Relatedly, an umbrella review including 14 meta-analyses indicated that RT produced medium-to-large effects on muscle strength and small-to-large effects on muscle power in youth [34]. A number of renowned stakeholders (e.g., National Strength and Conditioning Association, United Kingdom Strength and Conditioning Association, Canadian Society for Exercise Physiology, British Association of Sport and Exercises Sciences) have developed evidence-based position statements on the effects of RT on muscular fitness in youth [5, 14, 176, 198]. All these position papers concluded that RT has positive effects on muscular fitness in youth, irrespective of sex. Indeed, there is a widespread consensus in the literature that RT generates positive effects on muscular fitness in youth, regardless of sex, age and maturity [4, 5, 7, 9, 12, 44].

Although not as effective as RT, aerobic training may also improve muscular fitness. For example, aerobic and RT improved leg press strength (RT, 73%, from 60 to 86%; aerobic training, 42%, from 28 to 55%) compared with the control group in obese youth adolescents of both sexes [180]. A consistent trend was noted for bench-press, seated row, grip strength and push-ups (3–5% improvements and 5–12% improvements following aerobic and RT, respectively) [180]. Recent evidence indicates that lifelong RT can counteract the age-related denervation process and concurrent atrophy of type II muscle fibers in older male individuals [199]. This could contribute to promoting the maintenance of maximal strength and rate of force development, both of which are crucial for preserving functional capacity as individuals age [199]. On the other hand, individuals engaged in lifelong aerobic training displayed a lower proportion of type II muscle fibers and greater signs of atrophic fibers [199]. This suggests that early engagement in regular PA, particularly RT, is crucial for maintaining a high level of muscle strength and power in later life. These physical attributes play a pivotal role in sustaining functional capacity and independence as individuals age.

In summary, while aerobic training can offer certain benefits for muscular fitness, especially in individuals unaccustomed to regular PA, RT remains the gold standard method for achieving significant gains in muscular fitness in youth, particularly muscle strength and power.

### Neural and Muscular Adaptations

Although some degree of muscle hypertrophy can be anticipated during preadolescence [200, 201], neural adaptations during the prepubertal age usually take the lead over muscular ones in response to RT [12, 92, 173, 176, 192, 202–205]. For example, muscle strength and activation (assessed by surface electromyography and interpolated twitch technique) increased in prepubertal males and females without muscle size changes [201, 206]. The dominance of neural over muscular adaptations has largely been attributed to the low level of circulating anabolic hormones (e.g., testosterone) before maturation [207–209], although the central role of testosterone in this process has recently been questioned [210]. Once puberty takes place, muscular adaptations (i.e., muscle hypertrophy) in addition to neural ones (e.g., motor unit recruitment, rate coding) occur following RT [92, 211–214]. Therefore, differences in the magnitude of RT adaptations between pre-pubertal and post-pubertal individuals might occur. Indeed, Moran et al. [215] noted greater strength and power adaptations in postpubertal compared to prepubertal swimmers after 8 weeks of RT, hypothesizing fewer pathways of adaptation (mainly neural drive) in the prepubertal group compared to the postpubertal group (neural and morphological factors). However, the underlying mechanisms leading to different training-related adaptations between pre- and post-pubertal individuals need further exploration in similarly designed research studies. However, it is worth noting that an accelerated gain during puberty is not consistently observed across different components of muscular fitness, with variations between sexes [216]. Similarly, findings from a meta-analysis on the effects of RT according to age and maturation indicate that muscle strength increases with age and maturation, with no clear evidence of accelerated gains during puberty [213].

Of note, the heterogeneity of the assessment methods used in previous studies investigating prepubertal youth spanning from less accurate, reliable and sensitive measures (e.g., skinfolds, limb girth) to more sensitive and reliable ones (e.g., ultrasound, magnetic resonance imaging) could have hampered the interpretation of the outcomes [200]. As such, future studies in youth are needed that use more accurate methods to assess muscle hypertrophy following RT, to gain more comprehensive insights. Overall, it can be assumed that the dominance of neural adaptations during preadolescence will continue to prevail after RT, while the controversial results regarding muscular changes make it premature to conclude that this adaptation indeed takes place in this population.

## The Effects of Resistance Training and Aerobic Training on Cardiorespiratory Adaptations in Youth

Aerobic training is well accepted to be the gold standard mode of training to improve markers of cardiorespiratory fitness, such as VO_2max_ [217]. However, RT can also promote cardiorespiratory fitness. Indeed, following 3 months of training, Lee et al. [49] reported comparable cardiorespiratory fitness (i.e., VO_2peak_) improvements following aerobic (9.0 ± 0.9 ml/kg/min) and RT (7.6 ± 0.9 ml/kg/min) in obese male youth. However, in obese youth females, improvement was greater following aerobic training (5.17 ± 1.78 ml/kg/min) compared to RT (3.10 ± 1.69 ml/kg/min) [95]. Additionally, in obese youth of both sexes, 6 months of aerobic training improved VO_2peak_ to a greater extent (2.7%) compared to RT (0.9%) [180]. Sammoud et al. [186] reported increased endurance performance (20-m shuttle run test) after two modes of RT, plyometric training (d = 0.71) and power training using free weights (d = 0.95), in youth male soccer players. Similarly, Wong et al. [218] reported improved aerobic endurance (YoYo Intermittent Recovery Test total distance) after 12 weeks of combined strength and power training in youth male soccer players, in line with the findings of other studies in youth [219, 220].

Although the underlying mechanisms through which RT contributes to better cardiorespiratory fitness in youth have yet to be investigated, evidence in adults suggests that RT enhances running economy [221, 222], a key factor for endurance performance [223, 224]. In this regard, Wong et al. [218] revealed a significant reduction in submaximal running cost, indicative of improved running economy, which may be attributed to enhanced mechanical efficiency following combined RT and power training in youth. Additionally, increased tendon stiffness and enhancements in measures relating to neuromechanical factors (e.g., increased force-generating capacity) have been linked to better cardiorespiratory performance [225]. This is supported by earlier research demonstrating that RT improved both tendon stiffness and force-generating capacity [226, 227], facilitating a faster transfer of force from muscles to bones and reducing energy expenditure, ultimately contributing to a better running economy [228]. In summary, while aerobic training is the most effective method for improving cardiorespiratory fitness in youth, RT also has the potential to enhance cardiorespiratory fitness in this same population. It is worth noting that there are indications that combining aerobic exercise with RT (known as concurrent training) may produce a synergistic effect, resulting in enhanced cardiorespiratory fitness compared to either single-mode resistance training or aerobic training alone [220, 229]. However, this assumption lacked consistent reporting in the literature [230]. Therefore, future studies directly comparing the effects of aerobic and RT on youths' cardiorespiratory fitness are needed.

## The Era of Resistance Training as a Primary Form of Exercise for Health and Physical Fitness in Youth

Despite the dedicated efforts of various national and international health agencies, institutions, and scientists to disseminate the evidence highlighting the crucial role of PA for youth, the persistently low adherence rate to PA guidelines and the widespread trend of physical inactivity among youth raise serious concerns. While it is acknowledged that there is no single solution for such a multifactorial issue, optimizing PA guidelines by emphasizing the critical role of RT might contribute to promotion of youth physical fitness and health. Indeed, weaker youth often lack the physical ability, confidence and motivation to engage in exercise and sports activities, increasing the risk of adopting a sedentary lifestyle [7, 24, 54–56]. Early exposure to RT can help mitigate this risk by developing strength, confidence, and competence, providing a foundation for engaging in and maintaining regular PA while promoting adherence to PA guidelines [7, 9, 56].

**Fig. 1:**
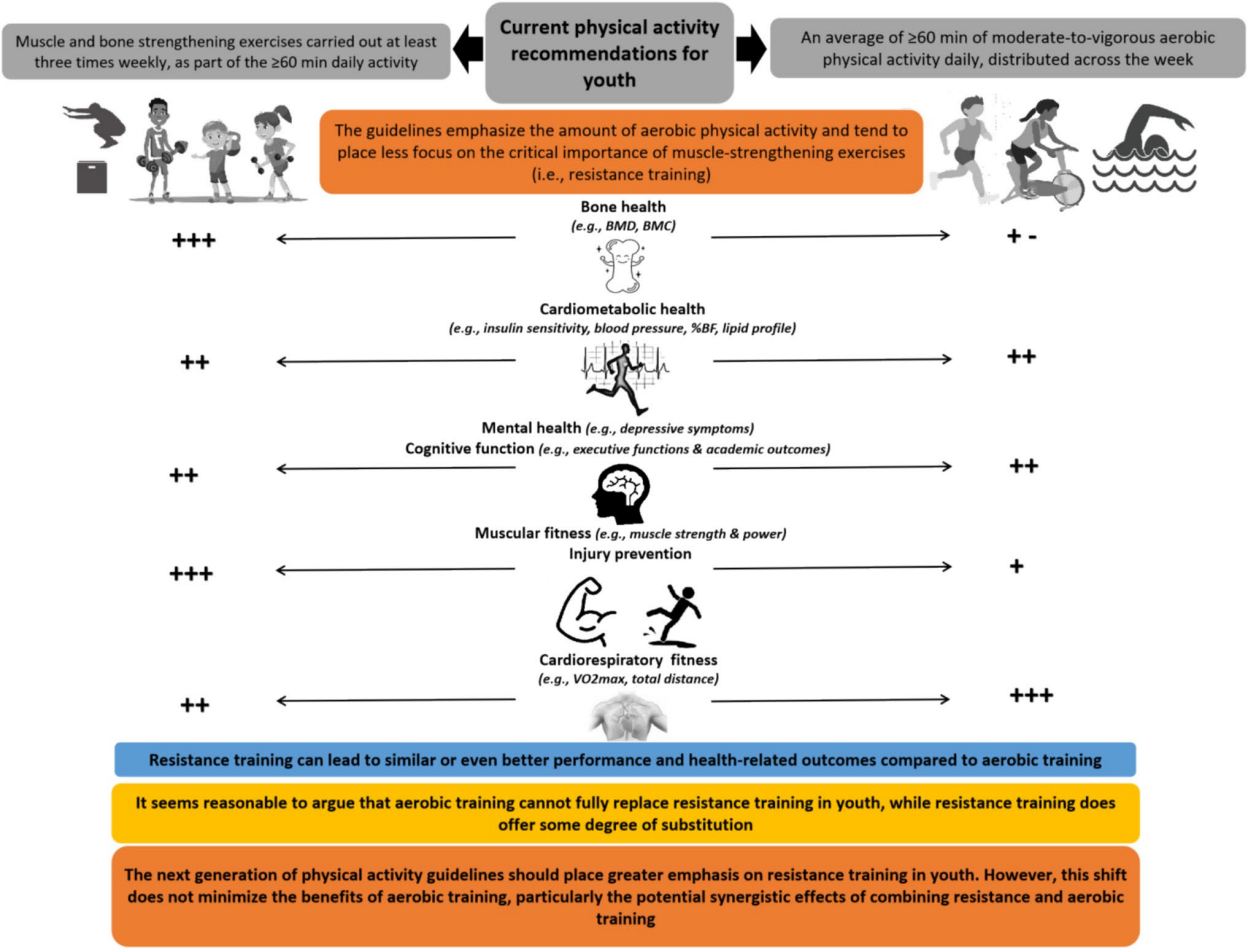
The effects of resistance training versus aerobic training on physical fitness and health in youth. The number of plus (**+**) symbols displays the magnitude of benefits of the respective component of physical activity. “**+ -**”: Indicates that the effects can be positive or neutral depending on the type of aerobic activity. *BMD* bone mineral density, *BMC* bone mineral content, *%BF* percentage of body fat, *VO*_*2max*_ maximal oxygen uptake

Emerging evidence suggests that RT may yield similar or even superior health benefits compared to aerobic training, including improvements in bone health, injury prevention, mental health, and cognition [6, 12, 54, 57, 114, 140, 141, 156, 157]. Additionally, RT has the potential to improve cardiorespiratory fitness [49, 95, 180, 186, 218], albeit to a lesser extent than aerobic training [95, 180] (Fig. 1). These findings suggest that, while aerobic training cannot fully replace RT in youth, RT may offer some degree of substitution. Therefore, we would argue that the traditional priority sequence, which apparently favours aerobic exercises over RT, should be reconsidered. In this vein, there seems to be an urgent need to update the current hierarchy of physical activity guidelines for youth by prioritizing RT and providing clearer recommendations on dosing. This shift does not minimize the benefits of aerobic training, particularly the potential synergistic effects of combining RT and aerobic training [48, 94, 97, 99, 100, 220, 229, 231, 232].

It is worth noting that performing RT exercises with proper techniques in youth is a top priority. While compelling evidence supports the safety of RT in this population [6, 205, 233], the risk of injury increases when technical proficiency is lacking, training loads are inappropriate, or supervision by qualified adults is absent [6, 93, 205]. Myer et al. [234] investigated RT-related injuries presented to US emergency rooms, with respect to age, type and mechanism. They concluded that the majority of youth RT injuries resulted from accidents that could potentially be prevented with improved supervision and the implementation of stricter safety guidelines [234]. In this sense, developing RT skill competency is essential, as it provides the foundation for safe and progressive training in a structured and appropriately challenging environment [4]. Therefore, youth must achieve sufficient technical mastery before advancing to more difficult and intense RT exercises [4]. More specifically, for youth with poor technical competency, the focus of a qualified practitioner should be on enhancing muscle strength while boosting their competence and confidence to perform various RT exercises [4]. On the other hand, technically competent youth can engage in more advanced RT exercises to optimize adaptations [4].

## Conclusions and Future Research Endeavors

RT is a well-established training approach to improve muscular fitness in youth. Of note, cumulative evidence indicates that the benefits of RT can also cover those traditionally attributed to aerobic training in youth. More specifically, there is evidence that RT improves cardiorespiratory fitness, decreases body fat, and increases insulin sensitivity, among others. Furthermore, in terms of bone health, there is persuasive evidence that RT, particularly plyometric training, yields substantial positive effects, whereas aerobic training may have a lesser or even neutral impact on BMD enhancement (e.g., swimming). Regarding mental health and cognition, while aerobic training has well-documented positive effects, preliminary evidence, albeit in obese youth, suggest that RT may offer even greater benefits. Moreover, it is well established that RT is a powerful means to decrease the risk and rate of injuries in youth. Overall, convincing evidence suggests that RT should not be considered secondary to aerobic training. Instead, it seems reasonable to consider RT as an essential (possibly even the most essential) aspect of PA in future national and international guidelines. While prioritizing RT, it is crucial to acknowledge the complementary benefits of combining RT with aerobic training. Given the severe detrimental effects of paediatric dynapenia, including the onset of functional limitations, physical inactivity, sedentary behaviour, and heightened susceptibility to various negative health outcomes, we contend that future guidelines should specifically emphasize RT.

Considering the relatively limited number of studies, future research should aim to directly compare the effects of RT and aerobic training on physical fitness and health in both unhealthy and, in particular, healthy youth. Additionally, to gain more comprehensive insights, there is a need to explore the effects of adhering solely to the current RT guidelines versus only to the current aerobic training recommendations on health and physical fitness in youth. Moreover, further studies are required to investigate the dose–response relationship of RT on physical fitness and health in youth. These research endeavours are crucial to provide better, more specific and detailed guidelines.

## Footnotes

1 Consists of jumping, hopping and skipping exercises that involve eccentric actions of the muscle–tendon unit, immediately followed by concentric actions – a process known as the stretch–shortening cycle [15].

2 Defined as a set of attributes that people have or achieve that relates to the ability to perform physical activity. It is categorized it into health-related (e.g., cardiovascular endurance, muscular strength, flexibility, body composition) and skill-related components (e.g., agility, balance, coordination, power, reaction time, speed) [16].

3 An insufficient physical activity level to meet current physical activity recommendations [44].

4 Activity in which the body’s large muscles move in a rhythmic manner for a sustained period of time. Aerobic activity – also called endurance activity – improves cardiorespiratory fitness. Examples include walking, running, swimming, and bicycling [44].

5 Fundamental movement skills refer to locomotor (running, skipping and hopping), manipulative (catching, throwing, grasping and striking), and stabilization (balance, twisting and bracing) skills [47].

6 Refers to the nervous system's ability to coordinate muscle activation for movement, force production and control.
